# Arbuscular mycorrhizal fungi mitigated nitrogen leaching by enhancing soil nitrogen retention in *Camellia oleifera* Abel. soils

**DOI:** 10.1128/aem.01487-25

**Published:** 2025-09-08

**Authors:** Fei Wu, Ziran Ma, Tuanjie Che, Yuxuan Huang, Ting Li, Na Wu, Xian Zhang, Linping Zhang, Xuetai Zhu, Xiaoling Zheng, Guoxiu Zhu, Rui Zhang

**Affiliations:** 1 College of Life Sciences, Northwest Normal University, Lanzhou, China; 2 Key Laboratory of State Forestry and Grassland Administration on Forest Ecosystem Protection and Restoration of Poyang Lake Watershed, Jiangxi Agricultural University 91595, Nanchang, China; 3 Innovation Center of Functional Genomics and Molecular Diagnostics Technology of Gansu Province, Lanzhou, China; 4 Institute of Applied Biotechnology, College of Agriculture and Life Science, Shanxi Datong University, Datong, China; University of Delaware, Lewes, Delaware, USA

**Keywords:** nitrogen leaching, arbuscular mycorrhizal fungi, dissolved total nitrogen, soil aggregates, *Camellia oleifera*

## Abstract

Nitrogen leaching is a major pathway of nitrogen fertilizer loss. Although arbuscular mycorrhizal (AM) fungi are known to reduce nitrogen leaching by improving plant nitrogen uptake, the soil-based mechanisms remain unclear. A pot experiment was conducted using a randomized complete block design, with four nitrogen levels (0, 3.75, 7.5, and 15 mM) and two AM fungal inoculation treatments. The study evaluated plant nitrogen uptake, hyphal length density, glomalin-related soil protein (GRSP) content, soil aggregation, soil nitrogen content, and nitrogen leaching loss in *Camellia oleifera* soils. Results showed that both NH_4_^+^-N and NO_3_^−^-N in leachate increased with increasing nitrogen application. AM fungi reduced NH_4_^+^-N (by 19.04% and 44.96%) and total nitrogen (TN, by 20.74% and 24.70%) in the leachate under high nitrogen conditions (7.5 and 15 mM). Under high nitrogen levels, AM fungi significantly increased soil easily extractable and total GRSP contents, NO_3_^−^-N, TN, and the proportion of 0.05–1.00 mm aggregates, while reducing soil dissolved total nitrogen (DTN) content, and the proportion of 0.002–0.05 mm aggregates. Redundancy analysis (RDA) showed that hyphal length density, DTN, soil NH_4_^+^-N, NO_3_^−^-N, and aggregate distribution were strongly associated with nitrogen leaching. Partial least squares path modeling indicated that soil DTN explained 96% of the variation in nitrogen leaching; AM fungi likely reduce soil DTN through increased soil aggregation via GRSP secretion and direct nitrogen absorption by extraradical hyphae. These findings highlight that changes in soil structure play a key role in reducing nutrient loss mediated by AM fungi.

**IMPORTANCE:**

Nitrogen fertilizers are essential for crop productivity, but low nitrogen use efficiency remains a major challenge in agriculture. Nitrogen leaching is a key contributor to nitrogen loss. Arbuscular mycorrhizal (AM) fungi are known to reduce fertilizer loss by enhancing nitrogen uptake in host plants. This study explored how AM fungi affect nitrogen leaching, plant nitrogen uptake, hyphal development, glomalin-related soil protein (GRSP) production, soil aggregation, and soil nitrogen content in *Camellia oleifera* soils. The results show that AM fungi can reduce nitrogen in soluble form and subsequently decrease nitrogen leaching, not only by facilitating nitrogen uptake via extraradical hyphae but also by altering soil structure—especially by promoting aggregate formation through the secretion of GRSP. The findings underscore that the functional significance of AM fungi should not be limited to their interactions with plant components, as their role in soil amelioration is equally important.

## INTRODUCTION

To meet the demands of a growing world population, food production relies heavily on mineral fertilizers, especially nitrogen (1). It is estimated that approximately 115 Tg of nitrogen fertilizers are applied worldwide, with 50%–70% of this nitrogen lost from agricultural systems to the environment (2). One significant pathway for nitrogen loss is leaching, which not only leads to the wastage of costly fertilizers but also contributes to water eutrophication, posing risks to human health and causing declines in biodiversity in downstream ecosystems (3, 4). Consequently, research on mitigating soil nitrogen leaching loss has been a long-standing focus.

While plant uptake is expected to be the main destination for nitrogen fertilizers, studies indicate that less than 50% of nitrogen applied is effectively absorbed by plants (2). In addition to plant uptake, soil microorganisms play a crucial role in minimizing nitrogen loss via leaching (4). Among these microorganisms, arbuscular mycorrhizal (AM) fungi form symbiotic relationships with the roots of approximately 80% of terrestrial plants (5). These fungi inhabit both the soil and plant roots, with intraradical hyphae growing within plant roots and enhancing the host’s nitrogen uptake capacity (6–8). Extraradical hyphae extend into the surrounding soil, broadening the root’s absorption range (9, 10). Research has shown that the mycorrhizal nitrogen uptake pathway can account for nearly 40% of a plant’s total nitrogen acquisition (11, 12). Many studies have demonstrated that AM fungi can reduce nitrogen loss via leaching (13–15). For instance, He et al. (13) found that AM fungi alleviated nitrogen leaching loss during rainfall events by enhancing nitrogen uptake in maize and lowering nitrogen concentrations in pore water (13). Similarly, Gou et al. (15) showed that AM fungi reduced nitrogen loss through increased plant nitrogen uptake and decreased runoff and sediment loss in agroecosystems (15). Studies using mutants lacking mycorrhizal associations as controls revealed that mycorrhizal rice exhibited improved nitrogen absorption and reduced nitrogen loss via leaching and runoff (16, 17). The primary mechanism through which AM fungi mitigate nitrogen leaching loss appears to be through enhancing plant nitrogen nutrition (13–15). Increased nitrogen content in plants can diminish the available nitrogen pool in the soil, thereby reducing nitrogen leaching (18). However, the reduction in nitrogen leaching attributed to AM fungi is not proportional to the increase in plant biomass and nitrogen content (18), indicating that the enhanced nitrogen interception by mycorrhizal plants cannot be fully explained by improved nitrogen uptake alone.

The extraradical hyphae of AM fungi also play a significant role in soil structure and function, which may further influence nitrogen loss via leaching (19, 20). AM fungi contribute to soil particle aggregation, a crucial factor affecting soil properties such as pore continuity, water retention capacity, and infiltration rates (21). The hyphae and glomalin-related soil protein (GRSP) secreted by AM fungi serve as binding agents that facilitate the formation of microaggregates (<0.25 mm) into macroaggregates (>0.25 mm) (22, 23). The positive impact of AM fungi on soil aggregation enhances the retention of water and nutrients within soil particles (15). Previous research has indicated that AM fungi can reduce cadmium and phosphorus leaching by increasing GRSP production and improving soil aggregation (24, 25). Therefore, the reduction in nitrogen leaching mediated by AM fungi may also be regulated by soil-related mechanisms. However, most studies have primarily focused on quantifying the reduction in nitrogen leaching and the enhancement of plant nitrogen uptake mediated by AM fungi, leaving the influence of AM fungi on soil physical and chemical properties—and their role in mitigating leaching—underexplored.

*Camellia oleifera* Abel. (Theaceae), recognized as one of the world’s four major woody oil crops, is predominantly distributed across China and Southeast Asian countries (26). Nitrogen fertilizer is the most crucial nutrient for *C. oleifera*, yet it is particularly vulnerable to leaching loss due to rainfall (27). *C. oleifera* can form symbiotic associations with AM fungi (28); however, the effects and underlying mechanisms of AM fungi on nitrogen leaching in this system remain largely unknown. This study aimed to elucidate the influence of AM fungi on nitrogen leaching loss in the *C. oleifera* system. We investigated the impacts of AM fungi inoculation on nitrogen leaching loss, plant nitrogen uptake, soil aggregate size distribution, GRSP content, and soil nitrogen content. We hypothesized that alterations in soil properties mediated by AM fungi represent a critical mechanism contributing to the reduction of nitrogen leaching losses.

## MATERIALS AND METHODS

### Fungal inoculum, plant material, and growth substrate

The AM fungus *Funneliformis mosseae* was provided by the Institute of Root Biology, Yangtze University (Hubei, China). The fungus was propagated with maize (*Zea mays*) in sterilized sand. The infected roots, spores (~50 spores g^−1^), mycelia, and cultured sand were mixed and used as AM inoculum.

Seeds of *C. oleifera* Abel. cultivar Changlin53 were collected from the Jiangxi Academy of Forestry Research, China. The seeds were surface sterilized for 10 min with 0.3% KMnO_4_ and rinsed three times with sterile water. They were then buried in sterilized sand in December 2020. Seedlings with uniform growth were selected for the experiment in May 2021.

Topsoil (0–20 cm) was collected from the campus of Jiangxi Agricultural University (Nanchang, China) and sieved with a 2 mm mesh. The soil physicochemical properties were as follows: pH (soil:water = 1:5) was 6.2, organic matter content was 22.35 g·kg^−1^, ammonium nitrogen (NH_4_^+^-N) content was 8.75 mg·kg^−1^, nitrate nitrogen (NO_3_^−^-N) content was 2.04 mg·kg^−1^, and available phosphorus content was 2.30 mg·kg^−1^. River sand was also sieved (2 mm) and thoroughly washed with tap water. The soil and sand were mixed at a 1:1 ratio and sterilized by autoclaving (121°C, 2 h), then used as the growth substrate.

### Experimental design and growth conditions

A pot experiment was conducted from May to October 2021 in a greenhouse at Jiangxi Agricultural University (Nanchang, China). The two-factor experimental design consisted of eight treatment combinations: AM treatment (inoculated with *F. mosseae* or not) and nitrogen treatment (0, 3.75, 7.5, and 15 mM N). These nitrogen concentrations were selected based on a previous study to represent levels ranging from nitrogen deficiency to excess (25). Each treatment had 20 replicates, resulting in a total of 160 pots. All pots were planted simultaneously in a single batch. Each *C. oleifera* seedling was planted in a plastic pot (17.0 cm top diameter, 12.0 cm bottom diameter, and 15.0 cm height) containing 2 kg of the growth substrate. A total of 80 pots were inoculated with 20 g of AM inoculum, while the remaining 80 pots received 20 g of autoclaved inoculum (121°C, 2 h) along with 10 mL of AM inoculum filtrate (<20 µm), which was obtained by resuspending the microbial pellet after centrifugation to avoid the influence of AM fungal metabolites. Seedlings were grown in a greenhouse maintained at 25°C–30°C with 30%–60% relative humidity under natural light conditions (14 h light/8 h dark). Temperature control was achieved using shade nets, water curtains, and ventilation fans, while humidity was regulated by a spraying system. All seedlings were adequately watered during the first 3 months. Afterward, AM-treated seedlings were divided into four groups of 20 and surface irrigated weekly with 200 mL NH_4_NO_3_ solution at concentrations of 0, 1.875, 3.75, and 7.5 mM NH_4_NO_3_. Nitrogen treatments were applied weekly for 10 weeks. All pots were maintained at field capacity throughout the experiment.

### Leachate collection and nitrogen content measurement

Prior to harvest, nitrogen treatments were applied at 17:00, and 300 mL of distilled water was added to each pot the following morning. Beakers were placed beneath each pot to collect leachate. After 30 min, most of the water was collected. The leachate volume was measured using a graduated cylinder and filtered through a 0.45 µm microporous membrane. The NH_4_^+^-N_leachate_ and NO_3_^−^-N_leachate_ concentrations were determined using a continuous-flow autoanalyzer (SKALAR-SA-40, Skalar Analytical BV, Breda, the Netherlands) (29). The total nitrogen (TN_leachate_) content was calculated as NO_3_^−^-N_leachate_ × leachate vol + NH_4_^+^-N_leachate_ × leachate vol.

### AM fungal colonization and hyphal length density

At harvest, the fresh fine roots were gently washed and stained with trypan blue (30). The AM fungal colonization rate was quantified using the magnified gridline intersection method under an optical microscope (Olympus BX43F, Tokyo, Japan) (31).

Soil samples were collected from the rhizosphere of the seedlings. AM fungal hyphal length density was determined using an aqueous extraction and membrane filter technique according to Jakobsen et al. (9). In brief, 2 g of soil was added to 250 mL of distilled water and blended for 30 s. Hyphae were collected from 15 mL of the suspension by filtering through 50 mm Millipore filters (1 µm pore size), stained with trypan blue for 5 min, and quantified under a microscope at 200× magnification using the intersection method.

### Glomalin-related soil protein

Easily extractable GRSP (EEG) and total GRSP (TG) were measured following the method of Wright and Upadhyaya (32). In brief, EEG and TG were extracted from 0.5 g of air-dried soil using 20 mM and 50 mM citrate solutions, respectively, at 121°C. The EEG and TG contents were measured at 595 nm using bovine serum albumin as a standard.

### Soil properties

Soil physicochemical properties were analyzed using standard soil testing procedures (33). In brief, soil organic carbon (SOC) content was measured by K_2_Cr_2_O_7_-H_2_SO_4_ oxidation method. The TN_soil_ concentration was measured using a Kjeltec 8400 Analyzer Unit (FOSS-Tecator, Hoganas, Sweden). Soil dissolved organic carbon (DOC) and dissolved total nitrogen (DTN) contents were measured by a total organic carbon (TOC) analyzer (MultiN/C2100 TOC/TN, Jena, Germany). Soil NO_3_^−^-N_soil_ and NH_4_^+^-N_soil_ contents were extracted using 2 mM KCl and measured with a continuous-flow autoanalyzer. Soil pH (soil:water = 1:5) was measured using a pH meter (PHS-3B, Jingke Inc., Shanghai, China). Soil aggregates were analyzed using a laser particle size analyzer (Mastersizer 2000, Malvern Instruments, Worcestershire, UK).

### Plant nitrogen content

The leaves, stems, and roots were harvested and oven-dried at 85°C to a constant weight to determine dry biomass. Samples were ground to a fine powder, and nitrogen concentration was measured from 0.5 g of sample using Kjeltec 8400 Analyzer Unit (34). The TN_plant_ content was calculated as leaf dry weight × leaf nitrogen concentration + stem dry weight × stem nitrogen concentration + root dry weight × root nitrogen concentration.

### Statistical analysis

Data analysis was performed using various packages in R version 4.0.2 (35). Normality and homogeneity of variances were tested using the shapiro.test() and bartlett.test() functions, respectively. A two-way analysis of variance (ANOVA) was used to assess the effects of AM treatment, nitrogen treatment, and their interaction on the measured parameters using the aov() function. An independent-samples *t*-test was conducted using the t.test() function to evaluate the significant differences between non-inoculated and inoculated plants at each nitrogen level. A one-way ANOVA followed by Tukey’s multiple comparison test was used to assess significant differences among nitrogen treatments using aov() and TukeyHSD() functions in the multcomp package at *P* < 0.05. The nitrogen leaching parameters (including NH_4_^+^-N_leachate_ and NO_3_^−^-N_leachate_ concentrations, and TN_leachate_ content) were analyzed using principal component analysis (PCA) via the prcomp() function. Redundancy analysis (RDA) was performed using the rad() function from the vegan package. Correlations between parameters were calculated using pairwise Pearson correlation coefficients with a Holm-corrected *P*-value. Partial least squares path modeling (PLS-PM) was conducted using the plspm package to assess the direct and indirect effects of soil parameters on nitrogen leaching loss (36). The model’s predictive performance was evaluated through the goodness of fit index (GoF) and coefficient of determination (*R*^2^). Figures were generated using the ggplot2 package.

## RESULTS

### AM fungal colonization and hyphal length density

**Fig 1:**
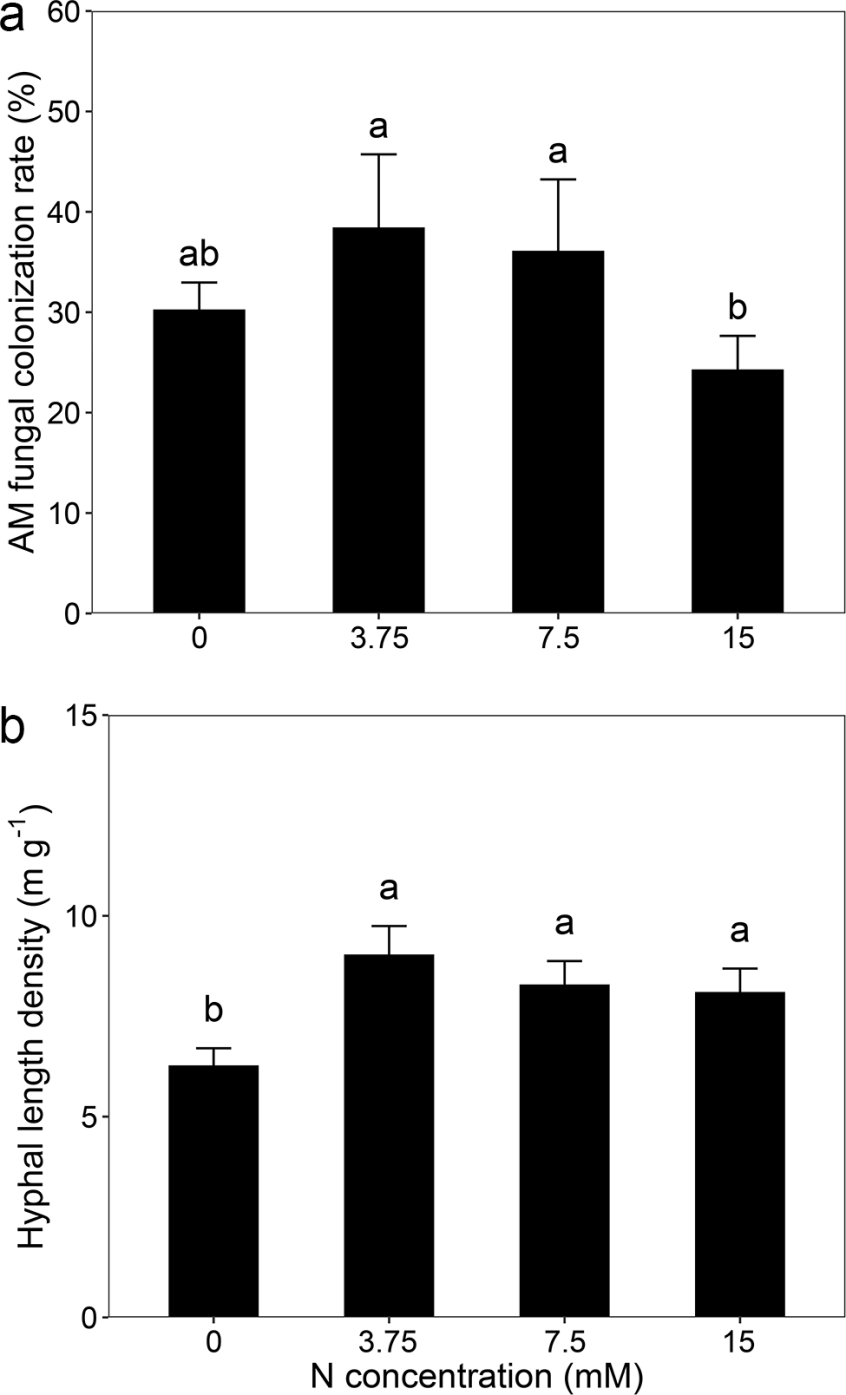
AM fungal colonization rate in the roots of *C. oleifera* (**a**) and hyphal length density in rhizosphere soil (**b**). Data are presented as means ± SD (*n* = 5). Different lowercase letters indicate a significant difference at *P* < 0.05 according to Tukey’s multiple comparison.

Nitrogen application significantly influenced AM fungal colonization and hyphal length density (Fig. 1). The colonization rate of AM fungi in inoculated plants exhibited an initial increase, peaking at 3.75 mM nitrogen, followed by a decline at higher nitrogen levels. Inoculated plants showed a significantly greater hyphal length density after nitrogen application compared to the non-fertilizer control. No AM structures were detected in non-inoculated plants.

### Nitrogen content in leachate

**Fig 2:**
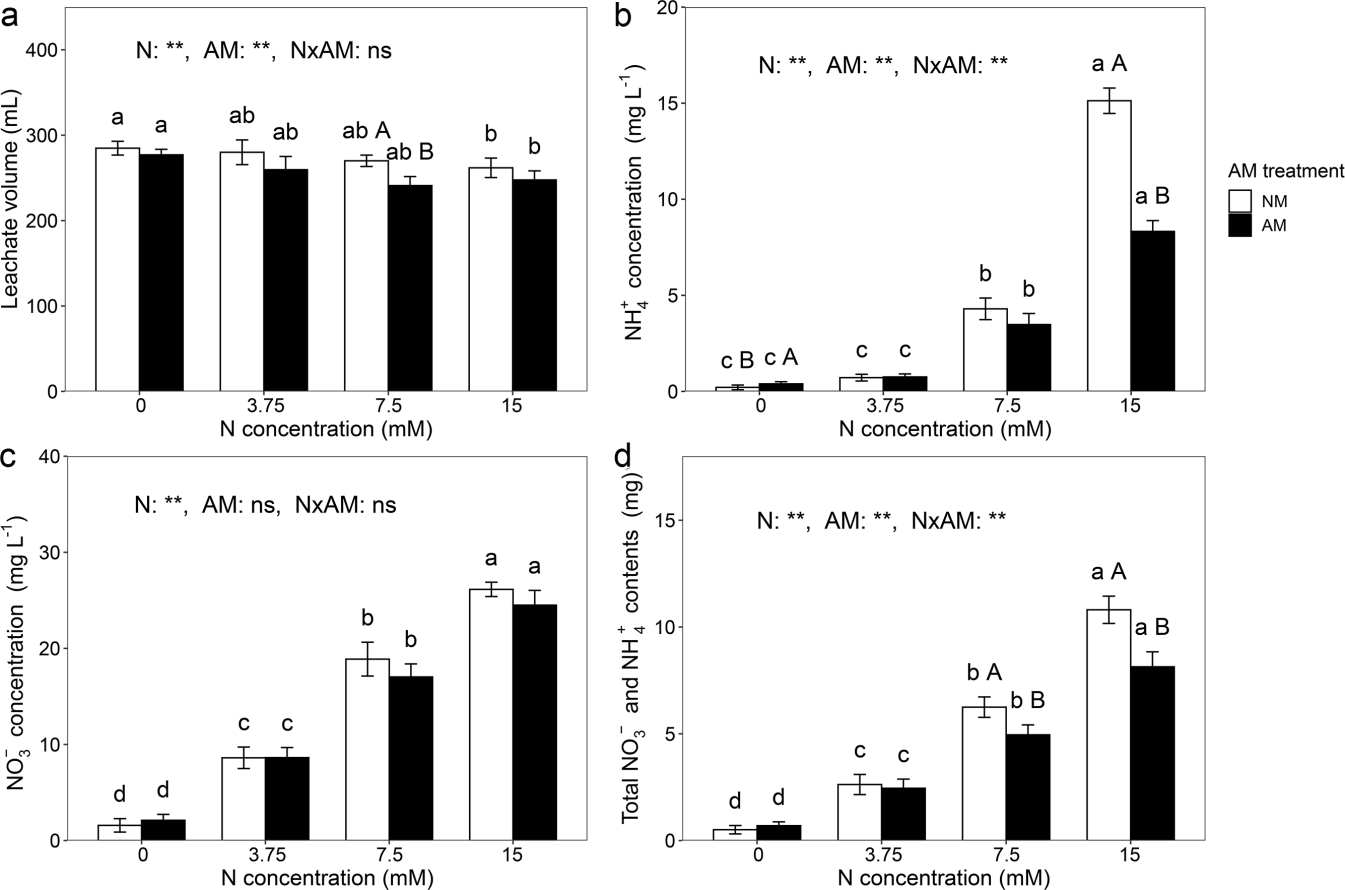
Leachate volume (**a**), ammonium nitrogen (NH_4_^+^) (**b**), and nitrate nitrogen (NO_3_^−^) concentrations (**c**), and total nitrogen (NO_3_^−^ and NH_4_^+^) content (**d**) in leachate. Data are presented as means ± SD (*n* = 5). Different lowercase letters indicate a significant difference among nitrogen levels under the same AM fungal inoculation treatment at *P* < 0.05 according to Tukey’s multiple comparison. Different uppercase letters indicate a significant difference between non-inoculated and inoculated treatments under the same nitrogen level at *P* < 0.05 according to independent-samples *t*-test. *: significant effect at 0.05 ≤ *P* < 0.01, **: significant effect at *P* < 0.01, and ns: no significant effect.

Both nitrogen and AM treatments significantly affected leachate volume, NH_4_^+^-N_leachate_ and NO_3_^−^-N_leachate_ concentrations, and TN_leachate_ content (*P* < 0.01, two-way ANOVA; Fig. 2). A significant interaction between nitrogen and AM treatments was also observed for NH_4_^+^-N_leachate_ concentration and TN_leachate_ content (*P* < 0.01, two-way ANOVA). As nitrogen application increased, leachate volume decreased, while contents of NO_3_^−^-N_leachate_ and NH_4_^+^-N_leachate_, and TN_leachate_ increased. Inoculated pots had lower leachate volumes (2.74%, 7.29%, 10.74%, and 5.42%) than non-inoculated pots across all nitrogen levels (0, 3.75, 7.5, and 15 mM nitrogen, respectively). In the absence of nitrogen, NH_4_^+^-N_leachate_ concentration was higher in inoculated treatments, but under 15 mM nitrogen, it was 44.96% lower in inoculated treatments. At 7.5 and 15 mM nitrogen, TN_leachate_ contents were significantly reduced by 20.74% and 24.70%, respectively, in the inoculated treatment compared to the non-inoculated ones. AM treatment did not significantly affect NO_3_^−^-N_leachate_ concentrations at any nitrogen level.

### Plant nitrogen content

**Fig 3:**
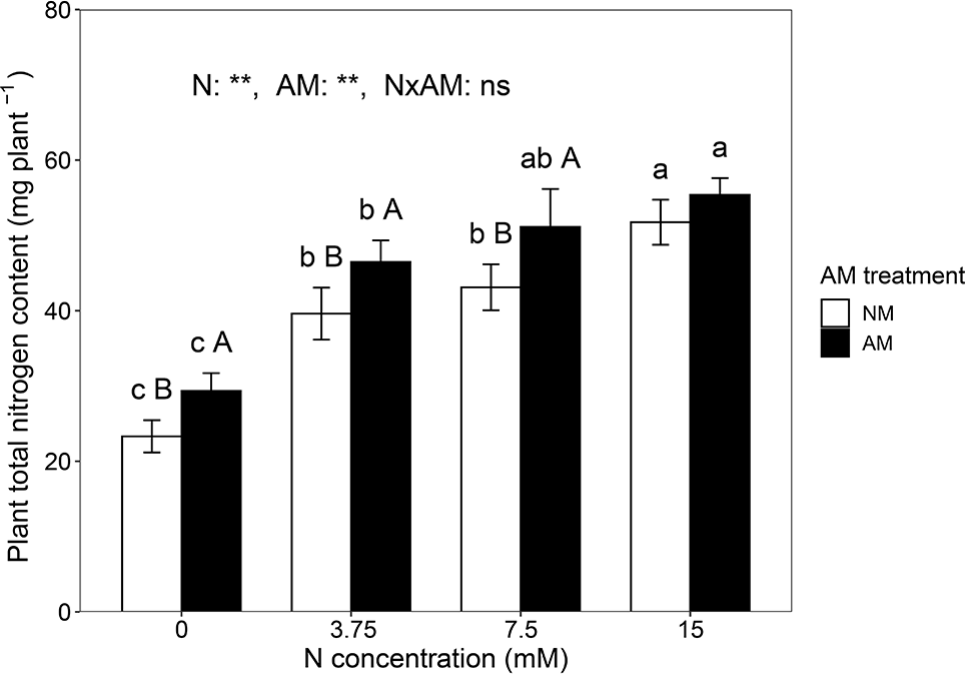
Plant total nitrogen content of *C. oleifera.* Data are presented as means ± SD (*n* = 5). Different lowercase letters indicate a significant difference among nitrogen levels under the same AM fungal inoculation treatment at *P* < 0.05 according to Tukey’s multiple comparison. Different uppercase letters indicate a significant difference between non-inoculated and inoculated treatments under the same nitrogen level at *P* < 0.05 according to an independent-samples *t*-test. *: significant effect at 0.05 ≤ *P* < 0.01, **: significant effect at *P* < 0.01.

The TN_Plant_ content was significantly influenced by both nitrogen and AM treatments (*P* < 0.01, two-way ANOVA; Fig. 3). Nitrogen application significantly increased TN_Plant_ content in both non-inoculated and inoculated plants. Inoculation with AM fungi enhanced TN_Plant_ content by 25.91%, 17.33%, and 18.59% under 0, 3.75, and 7.5 mM nitrogen, respectively.

### Soil properties

Two-way ANOVA revealed that nitrogen and AM treatments significantly affected soil DTN, NH_4_^+^-N_soil_, NO_3_^−^-N_soil_, TN_soil_, SOC, and DOC contents, and pH (excluding NH_4_^+^-N_soil_ under AM treatment; *P* < 0.01; Table 1). The interaction between nitrogen and AM treatment significantly influenced soil pH, DTN, and DOC contents (*P* < 0.01, two-way ANOVA). With increasing nitrogen levels, NO_3_^−^-N_soil_, NH_4_^+^-N_soil_, DTN, and TN_soil_ contents increased in both treatments. Inoculated treatments exhibited lower DTN content (68.39%, 55.11%, 42.21%, and 39.79%) and higher TN_soil_ content (12.23%, 10.44%, 9.43%, and 17.74%) compared to non-inoculated ones under all nitrogen levels. AM inoculation significantly increased soil NO_3_^−^-N_soil_ content under 15 mM nitrogen. No significant differences were observed in NH_4_^+^-N_soil_ content between treatments.

**TABLE 1:** Effects of nitrogen application and AM inoculation on soil properties^*a*^

Nitrogen treatment (mM)	AM treatment	DTN (mg kg^−1^)	NH_4_^+^-N (mg kg^−1^)	NO_3_^−^-N (mg kg^−1^)	TN_soil_ (g kg^−1^)	pH	SOC (g kg^−1^)	DOC (mg kg^−1^)
0	Non-inoculated	10.95 ± 1.19dA	6.08 ± 2.38c	4.39 ± 1.24d	0.47 ± 0.04b	5.17 ± 0.11aA	9.96 ± 0.55b	6.05 ± 0.57c
	Inoculated	3.46 ± 1.10cB	7.76 ± 3.33c	5.76 ± 2.24d	0.53 ± 0.06b	4.44 ± 0.19aB	10.24 ± 0.52b	6.38 ± 0.91d
3.75	Non-inoculated	18.04 ± 2.29cA	10.26 ± 3.29b	10.41 ± 1.60c	0.56 ± 0.05ab	5.15 ± 0.05bcA	10.86 ± 0.42ab	9.65 ± 1.79b
	Inoculated	8.10 ± 2.20bB	9.57 ± 3.38b	11.86 ± 1.77c	0.62 ± 0.05b	4.14 ± 0.04bB	11.54 ± 0.75a	9.19 ± 1.70c
7.5	Non-inoculated	31.65 ± 2.75bA	22.27 ± 3.84a	19.70 ± 2.86b	0.59 ± 0.08ab	5.04 ± 0.05cA	11.06 ± 0.43a	25.75 ± 1.07aA
	Inoculated	18.29 ± 2.39aB	18.37 ± 4.01a	23.11 ± 2.92b	0.64 ± 0.07ab	4.17 ± 0.03bB	11.76 ± 0.65a	22.2 ± 1.20aB
15	Non-inoculated	74.52 ± 2.38aA	68.22 ± 6.58a	28.31 ± 3.90aB	0.64 ± 0.08a	4.75 ± 0.06dA	11.13 ± 0.69a	24.39 ± 1.71aA
	Inoculated	44.87 ± 4.02aB	61.21 ± 4.12a	34.95 ± 3.47aA	0.75 ± 0.08a	4.04 ± 0.07bB	11.98 ± 0.76a	15.02 ± 0.90bB
Two-way ANOVA results								
*F*_N_		924.09**	441.13**	194.33**	14.84**	34.11**	11.67**	415.16**
*F*_AM_		382.05**	3.78ns	14.74**	11.89**	827.51**	10.71**	62.92**
*F*_N×AM_		41.76**	2.20ns	2.18ns	0.45ns	5.73**	0.40ns	28.62**

^*a*^ Data are presented as means ± SD (*n* = 5). Different lowercase letters indicate a significant difference among nitrogen levels under the same AM fungal inoculation treatment at *P* < 0.05 according to Tukey’s multiple comparison. Different uppercase letters indicate a significant difference between non-inoculated and inoculated treatments under the same nitrogen level at *P* < 0.05 according to an independent-samples *t*-test. **, significant effect at *P* < 0.01; ns, no significant effect. DTN: dissolved nitrogen content; NH_4_^+^-N: ammonium nitrogen content; NO_3_^−^-N: nitrate nitrogen content; TN_soil_: total nitrogen content.

Nitrogen application decreased soil pH and increased SOC content in both treatments. Soil DOC content initially increased and then decreased with rising nitrogen levels, peaking at 7.5 mM nitrogen in both treatments. Inoculated pots had lower soil pH (14.12%, 19.57%, 17.28%, and 14.98%) and higher SOC (2.86%, 6.33%, 6.31%, and 7.63%) compared to non-inoculated pots across all nitrogen levels. AM fungi reduced soil DOC content by 13.77% and 38.43% under 7.5 and 15 mM nitrogen, respectively.

### Soil aggregates

The proportions of soil aggregates (0.002–0.02 mm, 0.05–0.25 mm, and 0.25–1.00 mm) were significantly affected by nitrogen treatment, AM treatment, and their interactions (*P* < 0.05, two-way ANOVA; Table 2). Nitrogen application initially increased, then decreased the proportion of 0.002–0.02 mm aggregates, while it initially decreased, then increased the proportion of 0.05–0.25 mm aggregates. AM inoculation reduced the proportions of 0.002–0.02 mm aggregates (by 29.64% and 14.42%) and 0.02–0.05 mm aggregates (by 2.31% and 12.41%) under 7.5 and 15 mM nitrogen, respectively. Conversely, the proportions of 0.05–0.25 mm (by 122.63% and 44.93%) and 0.25–1.00 mm (by 47.44% and 34.18%) aggregates were significantly higher in inoculated soils at 7.5 and 15 mM nitrogen.

**TABLE 2:** Effects of nitrogen application and AM inoculation on the composition of soil aggregates^*a*^

Nitrogen treatment (mM)	AM treatment	Aggregate distribution (%)				
		<0.002 mm	0.002–0.02 mm	0.02–0.05 mm	0.05–0.25 mm	0.25–1.00 mm
0	Non-inoculated	1.39 ± 0.30	32.22 ± 3.25c	35.52 ± 4.38	24.43 ± 2.15a	6.45 ± 0.65a
	Inoculated	1.47 ± 0.38	35.54 ± 2.45b	35.58 ± 3.97	21.76 ± 3.34b	5.65 ± 1.08ab
3.75	Non-inoculated	1.68 ± 0.32	41.57 ± 3.59b	35.56 ± 2.78	16.51 ± 2.52b	4.68 ± 0.86b
	Inoculated	2.08 ± 0.47	40.44 ± 2.12a	33.13 ± 2.66	19.28 ± 1.52c	5.07 ± 1.15b
7.5	Non-inoculated	2.02 ± 0.43	49.16 ± 5.15aA	33.17 ± 3.93	11.16 ± 2.48cB	4.49 ± 1.01bB
	Inoculated	1.54 ± 0.41	34.59 ± 3.21bB	32.40 ± 3.61	24.84 ± 1.65abA	6.62 ± 0.51abA
15	Non-inoculated	1.39 ± 0.33	36.22 ± 4.35bA	38.76 ± 2.99A	18.42 ± 2.84bB	5.21 ± 0.74abB
	Inoculated	1.37 ± 0.37	31.00 ± 2.34bB	33.95 ± 2.50B	26.69 ± 1.63aA	6.99 ± 0.63aA
Two-way ANOVA results						
*F*_N_		4.32*	16.72**	2.06ns	14.48**	4.41*
*F*_AM_		0.00ns	16.27**	3.38ns	55.27**	10.38**
*F*_N×AM_		2.31ns	12.21**	0.99ns	22.51**	6.15**

^*a*^ Data are presented as means ± SD (*n *= 5). Different lowercase letters indicate a significant difference among nitrogen levels under the same AM fungal inoculation treatment at *P* < 0.05 according to Tukey’s multiple comparison. Different uppercase letters indicate a significant difference between non-inoculated and inoculated treatments under the same nitrogen level at *P* < 0.05 according to independent-samples *t*-test. *, significant effect at 0.05 ≤ *P* < 0.01; **, significant effect at *P* < 0.01; ns, no significant effect.

### Easily extractable GRSP and total GRSP

**Fig 4:**
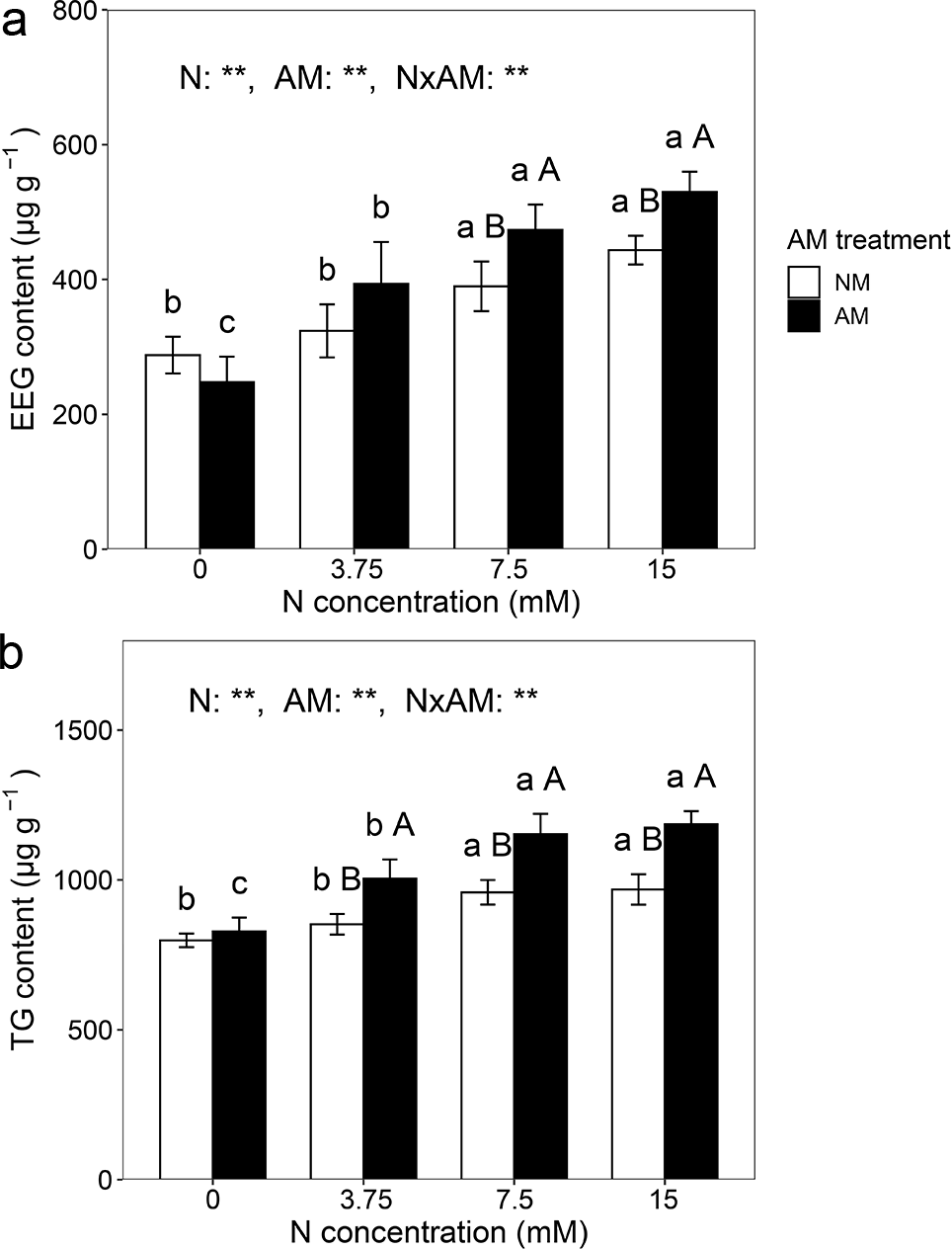
EEG (**a**) and TG (**b**) in rhizosphere soil of *C. oleifera.* Data are presented as means ± SD (*n* = 5). Different lowercase letters indicate a significant difference among nitrogen levels under the same AM fungal inoculation treatment at *P* < 0.05 according to Tukey’s multiple comparison. Different uppercase letters indicate a significant difference between non-inoculated and inoculated treatments under the same nitrogen level at *P* < 0.05 according to an independent-samples *t*-test. *: significant effect at 0.05 ≤ *P* < 0.01 and **: significant effect at *P* < 0.01.

Soil EEG and TG contents were significantly influenced by nitrogen treatment, AM treatment, and their interactions (*P* < 0.01, two-way ANOVA; Fig. 4). Nitrogen application increased EEG and TG contents in both non-inoculated and inoculated treatments. Under 7.5 and 15 mM nitrogen, EEG increased by 21.52% and 19.43%, and TG increased by 20.26% and 22.48%, respectively, in inoculated soils compared to non-inoculated soils.

### Relationship between AM fungal parameters, plant nitrogen uptake, soil factors, and nitrogen leaching loss

**Fig 5:**
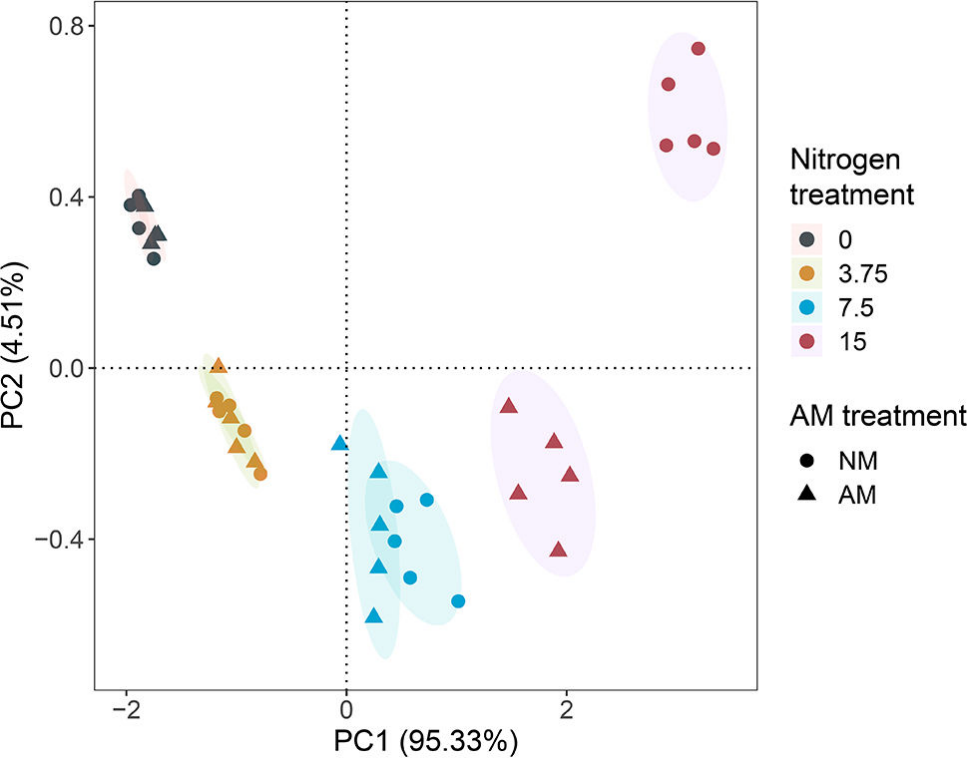
PCA plots of the variation in nitrogen content loss by leaching under different nitrogen and AM treatments.

PCA was conducted to analyze nitrogen leaching in response to nitrogen addition and AM inoculation (Fig. 5). The first two principal comments (PC1 and PC2) explained 95.33% and 4.51% of variance, respectively. In both non-inoculated (*F* = 102.84, *P* = 0.001, PERMANOVA) and inoculated (*F* = 121.72 *P* =0.001, PERMANOVA) treatments, samples were distinctly separated across the four nitrogen levels. Significant differences between non-inoculated and inoculated treatments were observed at 7.5 (*F* = 5.93, *P* = 0.013, PERMANOVA) and 15 mM (*F* = 53.88, *P* = 0.007, PERMANOVA) nitrogen.

Pearson correlation and RDA were performed to examine the relationships between leaching and plant, fungal, and soil variables at 7.5 and 15 mM nitrogen levels. Hyphal length density positively correlated with AM fungal colonization rate, SOC, EEG, TG, and the proportions of 0.05–0.25 mm and 0.25–1.00 mm aggregates, and plant TN_plant_ content, while negatively correlating with leachate volume, soil pH, DOC, and the proportion of 0.002–0.02 mm aggregates (Fig. S1). Leachate volume was positively correlated with soil DOC, pH, and the proportions of <0.002 mm and 0.002–0.02 mm aggregates, and negatively correlated with EEG, TG, and the proportions of 0.05–0.25 and 0.25–1.00 mm aggregates. The NH_4_^+^-N_leachate_, NO_3_^−^-N_leachate_, and TN_leachate_ contents were positively related to soil DTN, NH_4_^+^-N_soil_, and NO_3_^−^-N_soil_ contents, as well as the proportion of 0.02–0.05 mm aggregates.

**Fig 6:**
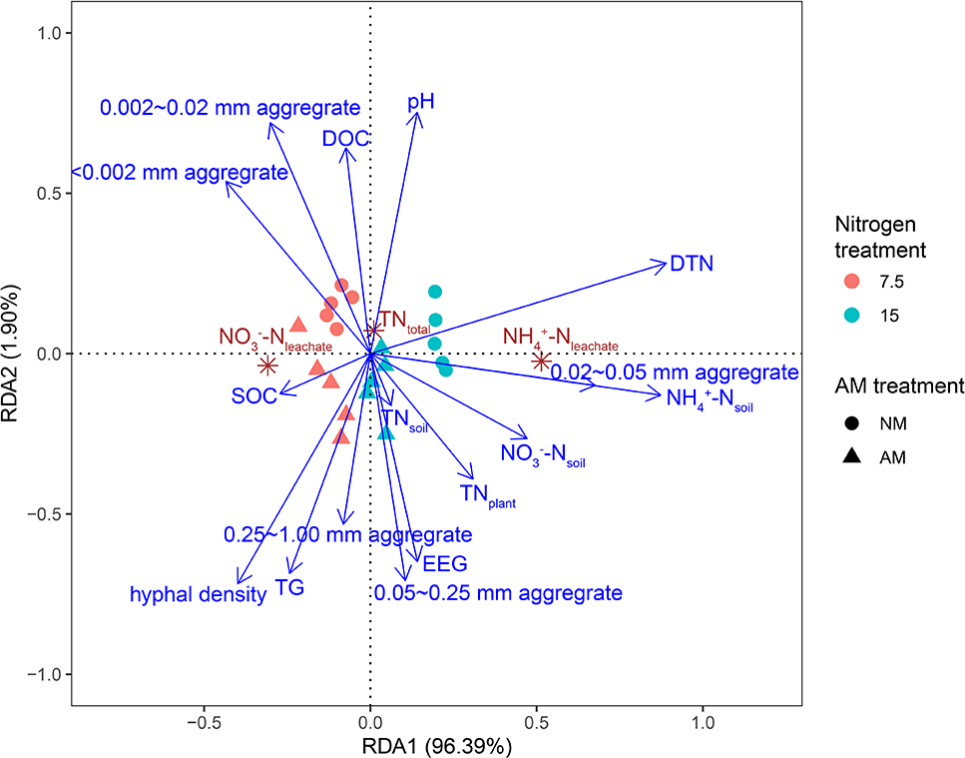
RDA showed potential factors that influenced nitrogen loss by leaching.

RDA indicated that plant, fungal, and soil factors explained 99.78% of nitrogen leaching variation, with RDA1 and RDA2 accounting for 96.39% and 1.90% of the total variance (Fig. 6). Hyphal length density, DTN, NH_4_^+^-N_soil_, NO_3_^−^-N_soil_, and the proportions of <0.002 mm and 0.02–0.05 mm aggregates were closely associated with leaching losses (Table S1).

**Fig 7:**
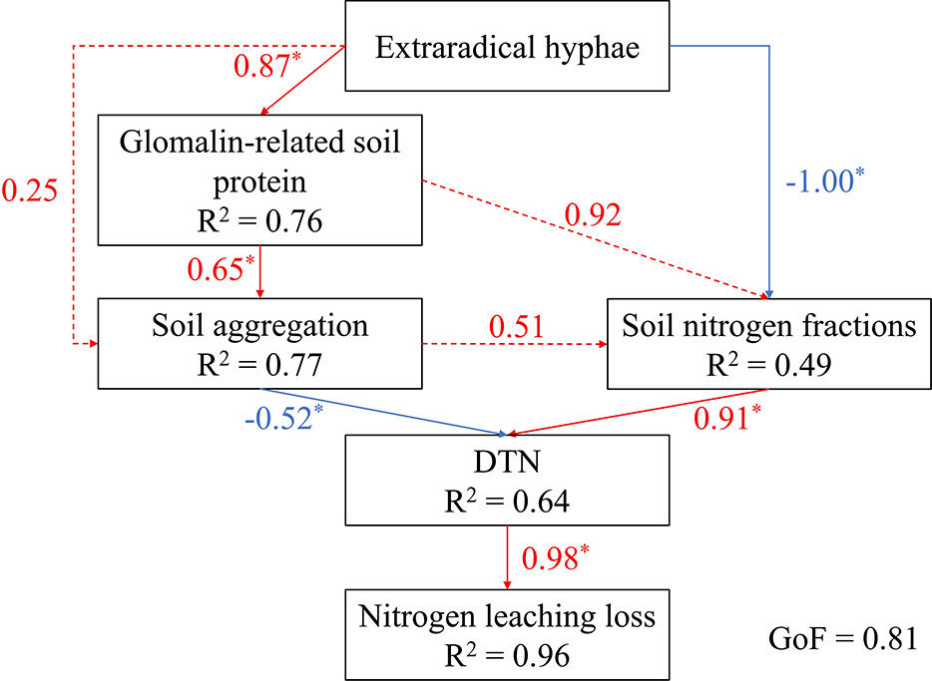
PLS-PM showing the relationships among the AM fungal parameters, soil properties, and nitrogen loss via leaching Note: Solid lines represent significant effects, and dashed lines represent non-significant effects. Positive and negative effects are represented by red and blue arrows, respectively. Path coefficients are shown as numbers near the lines. An asterisk indicates significant effects (*P* < 0.05). *R*^2^ indicates the proportion of variance explained by their independent latent variables.

A PLS-PM structural equation model (GoF = 0.807) was employed to explore the effects of AM fungi on nitrogen leaching (Fig. 7). Extraradical hyphae exhibited a direct positive effect on GRSP and a negative effect on soil nitrogen content. Although extraradical hyphae did not directly influence soil aggregation, they indirectly affected it via GRSP. Soil nitrogen positively influenced DTN, while soil aggregation negatively correlated with DTN. DTN had a strong positive influence on nitrogen leaching, explaining 96% of its variation.

## DISCUSSION

This study demonstrates that nitrogen leaching losses progressively increased with elevated nitrogen fertilization levels. AM fungi mitigated these nitrogen leaching losses, particularly under high nitrogen conditions. This mitigation effect is of paramount importance for agricultural systems, which frequently rely on excessive and uneven nitrogen fertilizer. While previous research has reported the alleviation of NH_4_^+^-N and/or NO_3_^−^-N losses by AM fungi across diverse ecosystems, including grassland (22, 37), agricultural fields (15, 38), and controlled pot experiments (18, 29), the underlying mechanisms largely remain elusive. This study provides new insights into the soil-mediated pathways by which AM fungi limit nitrogen leaching.

Consistent with previous studies (39), this study found that both NH_4_^+^-N_leachate_, NO_3_^−^-N_leachate_, and TN_leachate_ contents in leachate increased with nitrogen fertilization levels. The NO_3_^−^-N_leachate_ accounted for a significant proportion (61.96%–94.19%) of TN_leachate_, consistent with previous findings that NO_3_^−^-N is more mobile in soil and more readily leached than NH_4_^+^-N (37). In the inoculated treatments, AM fungi colonized 22.33%**–**42.40% of *C. oleifera* roots, leading to reductions in both leachate volume and total nitrogen leaching loss under 7.5 and 15 mM nitrogen. These results corroborated previous studies indicating that AM fungi can effectively reduce NH_4_^+^-N and/or NO_3_^−^-N losses, particularly under high nitrogen levels (29, 40). However, the extent to which AM fungi reduce the NH_4_^+^-N or NO_3_^−^-N leaching is variable. In this study, AM fungi primarily reduced total nitrogen leaching by limiting NH_4_^+^-N losses and reducing leachate volume, supporting the hypothesis that AM fungi preferentially absorb NH_4_^+^-N due to the additional energy required for the conversion of NO_3_^−^-N to NH_4_^+^-N (41). The observed smaller leachate volume in inoculated pots may reflect the role of AM fungi in enhancing plant transpiration and improving soil water retention (27, 42).

Mycorrhizal plants typically exhibit reduced nitrogen losses through increased plant nitrogen uptake (15). Mycorrhizal plants absorb nitrogen via two pathways: root direct pathway and the mycorrhizal pathway (6). The extraradical hyphae of AM fungi can extend over 10 cm beyond the root zone, accessing NH_4_^+^-N and NO_3_^−^-N and transporting them to the host, thereby expanding the effective absorption zone (10). The mycorrhizal pathway can contribute more than 40% of the total nitrogen to the host, enhancing its nitrogen uptake capacity (11, 12). In line with previous findings (14, 15), *C. oleifera* plants colonized by AM fungi exhibited higher nitrogen content under 0, 3.75, and 7.5 mM nitrogen. However, at 15 mM nitrogen, the impact of AM fungi was minimal, likely because the plants could independently absorb sufficient nitrogen (34). RDA results suggest that the reduction in nitrogen leaching under AM fungal inoculation was not primarily due to enhanced plant nitrogen uptake at high nitrogen levels (Fig. 6).

Soil physicochemical properties, including soil texture, pH, TN, and SOC, can influence nitrogen leaching (43). DTN, which includes soluble nitrogen in water and salt solutions, represents a direct source of nitrogen loss via leaching (42). To date, little is known about how AM fungi affect DTN levels. Our findings indicated that AM fungi inoculation reduced DTN content, thereby mitigating nitrogen loss. Pearson correlation analysis revealed a significant negative relationship between DTN and hyphal length density. Given that NH_4_^+^-N and NO_3_^−^-N are primary components of DTN (42), this reduction may be attributed to the direct uptake of these ions by AM fungi, as previous studies have identified multiple ammonium and nitrate transporters in AM fungi (e.g., GintAMT1, GintAMT2, and GintAMT3 in *Rhizophagus irregularis*) (44, 45). Stable isotope studies using ^15^N have confirmed that extraradical hyphae can absorb and translocate NH_4_^+^-N and NO_3_^−^-N to host plants (46, 47). Additionally, AM fungi growth consumes a considerable amount of soil nitrogen—approximately 3%–5% of fungal biomass (48, 49). Thus, the direct uptake of NH_4_^+^-N and NO_3_^−^-N by extraradical hyphae for fungal metabolism, coupled with subsequent translocation to the host, likely contributes to the reduction of DTN content and, consequently, mitigates nitrogen leaching losses.

While AM fungal inoculation reduced DTN, it concurrently increased NO_3_^−^-N_soil_ and TN_soil_ content. This suggests that AM fungi enhance nitrogen retention in the soil, particularly for NO_3_^−^-N_soil_ (29). Given the high mobility of NO_3_^−^-N_soil_ in soil, AM fungi may intercept soil nitrogen by modifying soil structure, as soil pore-size distribution influences water and nutrient retention (50, 51).

Soil aggregation plays a crucial role in soil structure development (52). AM fungi contribute significantly to soil aggregation through both physiological and chemical mechanisms (53). The hyphal length density of extraradical hyphae can exceed 10^8^ m/m^3^, promoting aggregate formation and stabilization through mechanical entanglement and adhesive properties (53, 54). Glomalin, a glycoprotein secreted by AM fungi hyphae, acts as a “soil glue,” facilitating the aggregation of microaggregates into macroaggregates (54). Numerous studies have emphasized the pivotal role of glomalin in soil aggregation and water retention (10, 55). In this study, AM fungi inoculation enhanced soil aggregation under high nitrogen conditions by increasing the proportions of 0.05–0.25 mm and 0.25–1.00 mm aggregates, while decreasing the proportions of 0.002–0.02 mm and 0.02–0.05 mm aggregates. This is consistent with previous findings that AM fungi promote the transformation of microaggregates into macroaggregates (22, 23). Pearson correlation analysis revealed positive correlations between TG and EEG contents with soil aggregation parameters. Furthermore, hyphal length density exhibited strong positive associations with the TG and EEG contents, as well as with the proportions of 0.05–0.25 mm and 0.25–1.00 mm aggregates. These results suggest that increased hyphal length density under higher nitrogen levels may contribute to greater glomalin production and improved soil aggregation (53, 54). The composition of soil aggregates substantially influences soil structure, water retention, and nitrogen storage capacity (56, 57). Previous studies have shown that macroaggregates typically contain higher nitrogen content than microaggregates (57, 58). In line with this, our findings revealed positive correlations between the proportion of 0.05–0.25 mm and 0.25–1.00 mm aggregates and TN_soil_ content. Under elevated nitrogen levels, AM fungi inoculation increased the abundance of macroaggregates, potentially enhancing nitrogen retention and thereby reducing nitrogen leaching losses (15, 51). This conclusion is further supported by the PLS-PM analysis, which demonstrated that AM fungi reduced soil DTN content by modifying soil structure and nitrogen dynamics, ultimately mitigating nitrogen leaching loss.

### Conclusion

AM fungi mitigated nitrogen leaching losses, with their ameliorative effects demonstrating dose-dependent responses to nitrogen application levels. AM fungi showed enhanced efficacy under high nitrogen fertilization conditions. At higher nitrogen levels, AM fungi reduced nitrogen leaching loss in *C. oleifera* soils primarily by enhancing nitrogen retention. AM fungi may decrease soil DTN content through two mechanisms: enhanced nitrogen interception via improved soil aggregation and direct uptake of soil nitrogen by fungal hyphae. These findings suggest that maintaining conditions conducive to AM fungal growth could be an effective strategy for sustainable nutrient management in future agroecosystems.

## Data Availability

All data supporting the findings of this study are available within the paper and its supplemental material.

## SUPPLEMENTAL MATERIAL

Supplemental material aem.01487-25-s0001.docx Fig. S1; Table S1.

ASM does not own the copyrights to Supplemental Material that may be linked to, or accessed through, an article. The authors have granted ASM a non-exclusive, world-wide license to publish the Supplemental Material files. Please contact the corresponding author directly for reuse.
