# Supplementary material for: Arbuscular mycorrhizal fungi mitigated nitrogen leaching by enhancing soil nitrogen retention in Camellia oleifera Abel. soils
Source: Appl Environ Microbiol. 2025 Sep 8;91(10):e01487-25. doi: 10.1128/aem.01487-25 (PMC12542650; doi:10.1128/aem.01487-25)
Supplement: Supplemental material — Fig. S1; Table S1. [file aem.01487-25-s0001.docx]

Arbuscular mycorrhizal fungi mitigated nitrogen leaching by enhancing soil nitrogen retention in *Camellia oleifera* Abel. soils

Fei Wu^1,2*^, Ziran Ma^1^, Tuanjie Che^3^, Yuxuan Huang^2^, Ting Li^1^, Na Wu^4^, Xian Zhang^1^, Linping Zhang^2^, Xuetai Zhu^1*^, Xiaoling Zheng^3^, Guoxiu Zhu^1^, Rui Zhang^1^

^1^ College of Life Sciences, Northwest Normal University, Lanzhou730070, China

^2^ Innovation Center of Functional Genomics and Molecular Diagnostics Technology of Gansu Province, Lanzhou730010, China

^3^ Key Laboratory of State Forestry and Grassland Administration on Forest Ecosystem Protection and Restoration of Poyang Lake Watershed, Jiangxi Agricultural University, Nanchang 330045, China

^4^ Institute of Applied Biotechnology, College of Agriculture and Life Science, Shanxi Datong University, Datong 037009, China


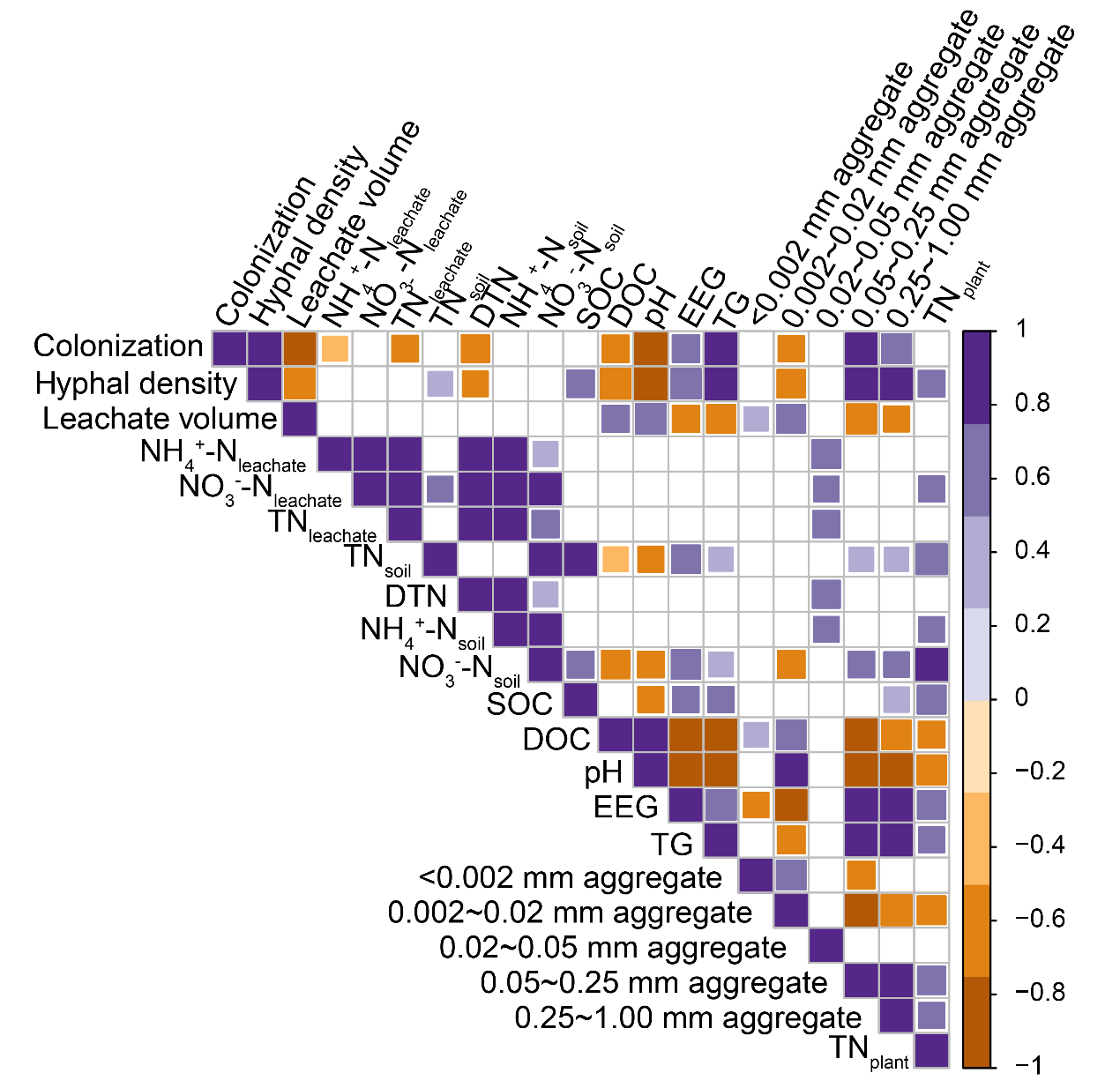


Fig. S1 Pearson correlations between AM fungi colonization, leachate parameters and soil parameters in rhizosphere of *Camellia oleifera*

Note: Only significant correlations were shown. The *P* value was corrected with Holm's correction method.

Table S1 The F and p-values after MANOVA results

| Indices | *F* | *P* |
| --- | --- | --- |
| Hyphal length density | 3.66 | **0.05** |
| DTN | 118.36 | **0.00** |
| NH_4_^+^-N_soil_ | 57.04 | **0.00** |
| NO_3_^-^-N_soil_ | 4.39 | **0.04** |
| TN_soil_ | 0.08 | 0.80 |
| pH | 0.52 | 0.50 |
| SOC | 1.37 | 0.26 |
| DOC | 0.24 | 0.64 |
| EEG | 0.40 | 0.52 |
| TG | 1.26 | 0.28 |
| <0.002 mm aggregate | 4.43 | **0.05** |
| 0.002~0.02 mm aggregate | 2.17 | 0.15 |
| 0.02~0.05 mm aggregate | 14.74 | **0.00** |
| 0.05~0.25 mm aggregate | 0.20 | 0.66 |
| 0.25~1.00 mm aggregate | 0.37 | 0.55 |
| TN_plant_ | 1.82 | 0.18 |

Note: DTN: dissolved nitrogen content, NH_4_^+^-N_soil_: soil ammonium nitrogen content, NO_3_^-^-N_soil_: soil nitrate nitrogen content, TN_soil_: soil total nitrogen content, SOC: soil organic carbon content, DOC: dissolved organic carbon content. EEG: easily extractable glomalin-related soil protein (GRSP) content, TG: total GRSP content, TN_plant_: plant total nitrogen content.
